# Hereditary angioedema due to C1-inhibitor deficiency: current therapeutic approaches

**DOI:** 10.1097/ACI.0000000000001042

**Published:** 2024-10-15

**Authors:** Giulia Costanzo, Giada Sambugaro, Davide Firinu

**Affiliations:** University of Cagliari: università degli studi di Cagliari, Monserrato, CA, Italy

**Keywords:** bradykinin, C1-inhibitor, hereditary angioedema, kallikrein, treatment

## Abstract

**Purpose of review:**

For decades, treatment options for hereditary angioedema (HAE) were limited by major adverse effects, insufficient efficacy, and difficult routes of administration. However, the growing body of knowledge regarding HAE pathophysiology has led to the development of innovative drugs for self-administered, on-demand therapy and for short- and long-term prophylaxis. This review provides a comprehensive overview of the approved drugs and the development of HAE treatments.

**Recent findings:**

The implementation of new therapies will improve the application of individualized action plans based on the key goals of minimizing the number of attacks and meeting the complex needs of patients.

**Summary:**

HAE is a rare genetic disease with a high impact on patients’ quality of life due to the unpredictability and variable severity of attacks. Advances in HAE research have allowed optimization of attack management and individualization of therapeutic approaches.

## INTRODUCTION

**FIGURE 1:**
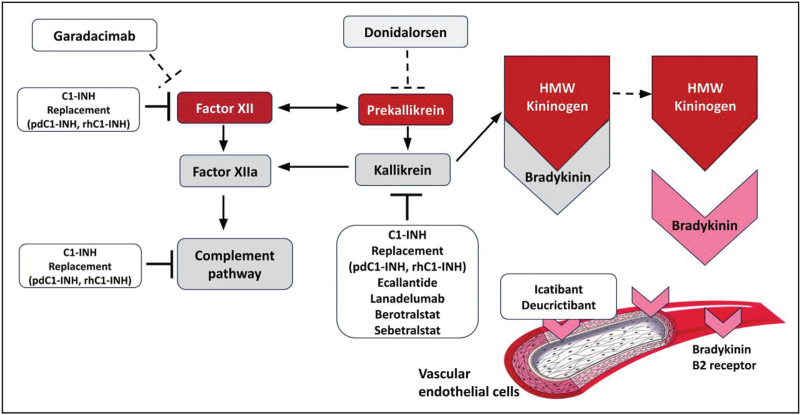
C1-INH regulates the activation of the complement system, coagulation, fibrinolysis, and contact system by inhibiting the activation of factor XII (FXII) and kallikrein. After the activation of the contact system (e.g., by negatively charged surfaces or trauma) and the formation of activated FXII, prekallikrein is converted to kallikrein, which releases bradykinin (BK) from high-molecular-weight kininogen (HMWK) cleavage. Plasmin, which is generated from plasminogen, enhances the activity of kallikrein in releasing BK from HMWK. BK in turn binds to its specific receptor B2 at the endothelial level, causing an increase in vaso-permeability and vasodilation and thus leading to the formation of angioedema.

Hereditary angioedema (HAE) is a rare genetic disease characterized by transient and recurrent attacks of edema in the subcutaneous tissue, mucosa, and submucosa, which are not accompanied by itching or swelling and are caused by a temporary and localized increase in endothelial permeability [1]. HAE is most frequently caused by mutations in the *SERPING1* gene, which are usually transmitted as an autosomal dominant trait, and in 25% of the cases, the disease is caused by a de novo mutation that results in congenital deficiency of complement inhibitor 1 (C1-INH). HAE caused by C1-INH deficiency (C1-INH-HAE) is classified as type 1 (reduced antigenic plasma levels) or type 2 (dysfunctional C1-INH at normal or high levels) [2,3]. C1-INH regulates the activation of the contact system by inhibiting the activation of factor XII (FXII) and kallikrein that generate bradykinin (BK), which binds to its specific receptor B2 at the endothelial level to enhance vaso-permeability and vasodilation and cause the formation of angioedema (Fig. 1) [4]. 

**Box 1:**
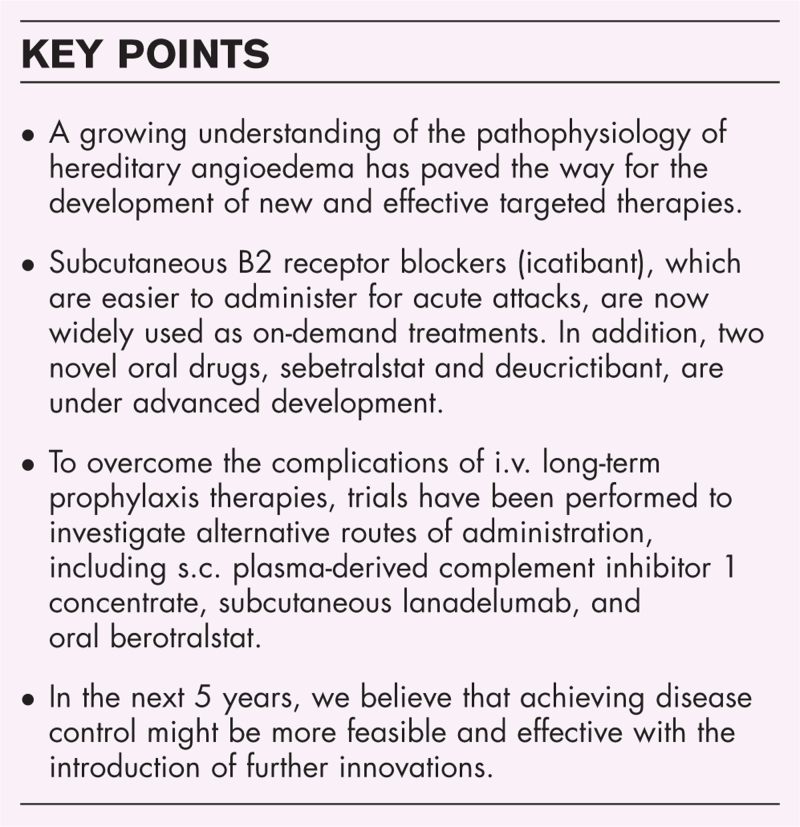
no caption available

The clinical onset of HAE generally occurs during childhood or adolescence [5] and can potentially affect all districts, but mostly affects the skin, gastrointestinal tract, and upper airways [5]. Some angioedema attacks are preceded by prodromic symptoms and signs such as fatigue, a tingling sensation, or a skin rash without itching that may be mistaken for an allergy, called erythema marginatum [6,7]. The attacks may be very painful, disfiguring, and associated with functional impairment (e.g., attacks on the extremities), leading to a strong impact on the patients’ quality of life (QoL) [8^▪^]. Without specific treatment, the attack can last 2–5 days and may be associated with mortality (e.g., life-threatening episodes of laryngeal edema) [9]. HAE should be suspected in patients with a history of recurrent angioedema without urticaria, positive family history, episodes of laryngeal edema, and recurrent abdominal pain that does not respond to antihistamine therapy, adrenaline, and/or glucocorticoids. Considering these clinical factors, diagnosis should be confirmed by laboratory tests assessing the levels of C4 and quantitative and functional C1-INH [10]. Patients with HAE type 1 show a reduction in the levels of C4 and quantitative and functional C1-INH, while those with HAE type 2 show normal or increased, but functionally reduced (<60%), levels of C1-INH. In HAE with normal C1-INH levels, the levels of antigenic and functional C1-INH and antigenic C4 are normal. Genetic tests for mutations responsible for HAE with normal C1-INH levels should be performed in suggestive clinical settings [11].

### Therapeutic approach

**FIGURE 2:**
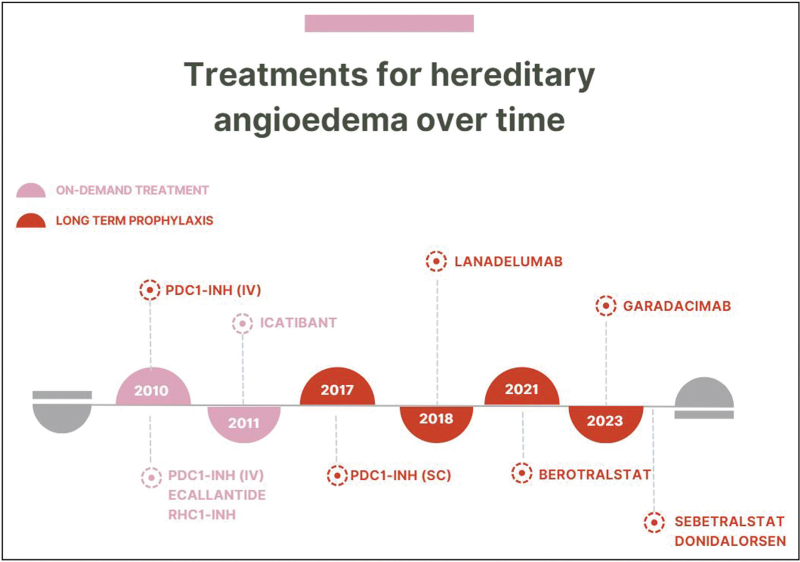
Several new therapies have been developed in recent years due to the improved knowledge of HAE pathophysiology, yielding medications that are safer, more effective, and easier to administer. Since the concept of prophylaxis has been introduced, specific drugs have been developed and approved, including subcutaneous plasma-derived C1 inhibitors, lanadelumab, and berotralstat. HAE, hereditary angioedema.

The improved understanding of HAE pathophysiology has led to the development of several new therapies, thereby creating safer, more effective, and easier medications, as shown in Fig. 2.

The existing pharmacological approach for C1-INH-HAE is based on three principles: on-demand treatment for acute attacks, which is indicated in all patients for all attacks on the basis of the patient's decision and should be administered as soon as possible; long-term prophylaxis (LTP), which is performed for patients experiencing frequent, disabling, severe attacks that cause strong impairment of the patients’ QoL; and short-term prophylaxis (STP), which is used for prevention of attacks in predictable situations [10]. Educating patients about the potential triggers of attacks and measures to avoid them is important. Moreover, early or prodromic symptoms should be recognized to promptly manage an acute attack, considering the correlation between early treatment and increased effectiveness [10,12]. Each patient should have a specific treatment plan based on their characteristics and frequency of attacks, which can vary in frequency, severity, and location throughout life. Shared decision-making may be an optimal approach [13,14,15^▪^]. Several effective on-demand treatments have become available over the last decade, but patients may still experience a substantial disease burden, especially those showing a high frequency of attacks [16,17].

### On-demand treatment

HAE attacks can be very painful, life-threatening, and/or associated with complications. Treatment should be considered promptly in all attacks, and is mandatory for attacks with upper airway involvement [10]. The administration of exogenous C1-INH directly but transiently replenishes C1-INH deficiency and has become a cornerstone therapy since the 1980s [18,19]. Starting in 2009, pasteurized plasma-derived C1-INH concentrate was licensed for intravenous (i.v.) administration, at-home treatment, and for self-administration [20]. i.v. C1-INH concentrate has been shown to be effective in treating patients with HAE type 1 or type 2 at all sites. The IMPACT-1 trial demonstrated that 20 U/kg of i.v. C1-INH concentrate could relieve symptoms in patients experiencing acute attacks faster than a placebo (0.5 vs. 1.5 h). The IMPACT-2 study confirmed that in-demand treatment with C1-INH concentrate was effective, with symptom relief starting within 30 min [21,22]. A nano-filtered plasma-derived C1-INH (pdC1-INH) concentrate was authorized by the European Medicines Agency (EMA) in 2011 for the treatment of HAE in adults at a dose of 1000 Units i.v. as soon as HAE attacks appeared and repeated after an hour if necessary [23]. Intravenous administration of recombinant human C1-inhibitor (rhC1-INH) has also been demonstrated to be safe and effective in resolving angioedema attacks at a dose of 50 IU/kg for patients weighing <84 kg and 4200 IU for those weighing >84 kg [24].

The last decade has seen the development of new therapies for acute attacks that are easier to administer via the subcutaneous route (facilitating prompt use outside healthcare settings): B2 receptor blockers and first-generation kallikrein inhibitors. Icatibant was the first subcutaneous B2 antagonist licensed by the EMA in 2008 and the US Food and Drugs Administration (FDA) in 2011 as an on-demand medication for patients aged at least 2 years [25]. A randomized DBPC study found that icatibant significantly improved the median time to symptom severity reduction and onset of symptom relief in comparison with placebo [26]. It is administered subcutaneously and can be easily self-administered at home by patients or caregivers at a dose of 30 mg (at most three injections within 24 h) [26]. Ecallantide, a recombinant inhibitor of plasma kallikrein for subcutaneous use registered for patients >12 years, received FDA approval in 2009 at a dose of 30 mg, and is administered as three subcutaneous injections by a healthcare professional (owing to the risk of anaphylaxis), but it did not receive EMA approval [27]. Sebetralstat is a novel oral kallikrein inhibitor [28,29] that is rapidly absorbed and leads to near-complete kallikrein inhibition within 20–30 min that is maintained for up to 8 h [29]. In a phase 2 trial, three oral administrations of 600 mg of sebetralstat were well tolerated, leading to rapid inhibition of plasma kallikrein activity and resulting in faster symptom relief than placebo, as measured by the Patient Global Impression of Change (PGI-S) score. It caused no serious adverse events or discontinuations due to adverse events [30]. Sebetralstat was evaluated in a randomized DBPC phase 3 crossover trial (KONFIDENT) for on-demand treatment of adolescent and adult patients with C1-INH-HAE [29]. Time to symptom relief using the Patient Global Impression of Change (PGI-C) scale was selected as the primary endpoint, based on patient preference [31]. In comparison with a placebo, sebetralstat provided significantly faster symptom relief, reduced attack severity, and yielded complete attack resolution [32^▪▪^]. Another therapeutic approach involves the use of deucrictibant (PHA-022121), a novel small molecule under investigation for LTP and on-demand treatments. Deucrictibant is an orally administered, specific antagonist of B2R. Rapid exposure and predictable linear pharmacokinetics at doses of up to 50 mg were demonstrated using data from a phase 1 study in healthy volunteers [33]. A phase 2 trial assessed the efficacy of oral deucrictibant for the treatment of acute attacks in patients with HAE. In Part II of the study, three different doses of deucrictibant and placebo were randomly assigned to eligible subjects at home to treat three HAE attacks. In the RAPIDe-1 phase 2 trial, deucrictibant treatment showed a rapid onset of action, fast time to symptom relief, and resolution of HAE attacks in comparison with placebo and was well tolerated [34].

In the current clinical practice, the management of patients with an acute episode of angioedema, is influenced by the patient's history, clinical manifestations and availability of therapies. In the absence of a previous diagnosis of HAE, even in the strong suspicion of C1-INH-HAE in an emergency setting, the first line therapy should first involve when appropriate the administration of antihistamines, corticosteroids, and/or adrenaline (anaphylaxis). The ineffectiveness of these drugs may strengthen a suspicion that has been prompted by the patient's medical history. The emergency doctor will evaluate the administration of an i.v. C1-INH formulation, icatibant or ecallantide as first line therapy for C1-INH-HAE as recommended by the international guidelines for HAE. The approach will be different for the patient with a confirmed diagnosis of HAE. In this case, the patient may usually bring the medication prescribed by the specialist to the Emergency Room, almost always accompanied by a letter with instructions and usage guidelines. In this case, the medication recommended by the specialist should be quickly administered. If the patient does not have any medication for acute attacks with him, the emergency room doctor may usually choose between the use of the C1-INH or icatibant. Since the last decade, many patients treat their attacks at home, by self-administration or supported by a caregiver.

However, targeted therapies are often expensive and/or unlicensed in many low- and middle-income countries or not available in the emergency rooms of hospitals not linked to an HAE Center. In this setting the only available solution, is represented by the administration of fresh-frozen plasma that can rapidly replace the C1-INH deficiency.

### Short-term prophylaxis

STP is recommended before procedures that can trigger an angioedema attack [10], typically prior to dental, surgical, or medical procedures, or in cases involving patient-specific known triggers. i.v. pdC1-INH concentrates are an effective and safe option for STP. C1-INH concentrates have been shown to supplement C1-INH deficiency. The nano-filtered C1 inhibitor concentrate has been approved for STP in HAE at an i.v. dose of 1000 U administered within 24 h before the procedure [23]. The pasteurized i.v. pdC1-INH concentrate has also been approved for STP at a dose of 1000 U and is administered within 6 h before the procedure [20]. Despite the appropriate use of STP, angioedema attacks can still be triggered by a procedure. Therefore, patients should be monitored after the procedure, and on-demand treatment should be available in case of an attack [10].

### Long-term prophylaxis

The goal of LTP is to reduce the frequency, severity, and duration of recurrent angioedema. For many years, nonspecific drugs such as attenuated androgens and antifibrinolytics were used off-label with low efficacy or uncertain risk/benefit ratios [35,36]. However, the subsequent development of therapies specifically targeting key pathways has facilitated on-label, safe, effective, and convenient administration of medications. i.v. pdC1-INH was approved by the FDA and EMA for use in LTP at a dose of 1000 U every 3–4 days [23]. It was shown to decrease the frequency, duration, and severity of angioedema attacks, as well as the need for acute rescue treatment. Regular use of i.v. pdC1-INH has also been reported to increase health-related QoL [37]. However, a patient survey showed that approximately 20% of patients receiving i.v. pdC1-INH had disruptive angioedema attacks once a month, and more than 10% experienced attacks 2–3 times a week [38]. To avoid this, the dose and interval of i.v. pdC1-INH administration should be individualized, with the lowest interval between doses being 48 h. In one study, patients with one attack/month were eligible for an increase in the dose of i.v. C1-INH (1500 IU, 2000 IU, and 2500 IU twice a week), and most patients showed a reduction in the frequency of attacks and good tolerance [39]. Although i.v. access is required for administration, many patients can be trained in self-administration [40]. To overcome peripheral venous access depletion and the risk of thrombotic events secondary to the use of indwelling catheterization [41], additional products such as subcutaneous pasteurized plasma-derived C1-INH concentrate (s.c. pdC1-INH) have been developed. Subcutaneous pdC1-INH was licensed by the FDA in 2017 for use in adolescents and adults at a dose of 60 IU/kg twice weekly as LTP. Its clinical efficacy was demonstrated in the phase 3 COMPACT trial. The proportion of patients showing <1 time-normalized HAE attacks per 4 weeks was 100% in the subgroup aged ≤17 years and 84.5% in the subgroup aged >17 years [42]. In the long-term extension arm of COMPACT, 83% of patients receiving the recommended dose were free of attacks, and treatment improved HAE-related QoL [43]. s.c. pdC1-INH has also demonstrated long-term safety and efficacy in both children and older patients with HAE [44–46]. Lanadelumab is a subcutaneous fully human immunoglobulin G1κ (IgG1κ) monoclonal antibody directed against plasma kallikrein that was approved by the FDA and the EMA for LTP in patients aged ≥12 years [47]. The phase 3 HELP study showed that among patients with C1-INH-HAE treated with subcutaneous lanadelumab for 26 weeks, the average breakthrough attack rate was significantly lower in the groups that received 300 mg every 2 and 4 weeks than in the placebo group. The current recommended dose is 300 mg every 2 weeks; however, a tentative prolongation of the dosing interval to 4 weeks can be considered once clinical remission is achieved [48]. The HELP-OLE study showed that lanadelumab demonstrated sustained efficacy and tolerability with long-term use in patients with HAE [49]. Berotralstat, a small-molecule drug that selectively inhibits plasma kallikrein and is administered orally once daily, is the first targeted oral prophylactic agent available for HAE that has been approved by the FDA and EMA for patients aged 12 years and older [50]. In 2016, a phase 1 trial showed that berotralstat had a half-life of 50–60 h and reached therapeutic plasma concentrations in 7 days without major safety issues. Then, a phase 2 trial found that berotralstat reduced the number of angioedema attacks up to 73.8%, with secondary endpoints supporting its efficacy at doses of ≥125 mg [51]. The APeX-2 randomized DBPC phase 3 study demonstrated a significant reduction in the attack rate at both 110 mg and 150 mg daily [52]. The proportion of patients who experienced 50% or greater reduction during the 24 weeks of the study was 25% in the placebo group, 51% in the 110-mg berotralstat group (odds ratio (OR): 3.042 [95% confidence interval (CI): 1.183–7.821]; *P* = 0.021), and 58% in the 150-mg berotralstat group (OR: 3.913 [95% CI: 1.507–10.164]; *P* = 0.005) [52]. In that study, the most frequent treatment-emergent adverse events were abdominal pain, vomiting, diarrhea, and back pain. No serious drug- or treatment-related adverse events were observed. The most favorable risk-benefit profile was observed at 150 mg/day [52].

Garadacimab was the first subcutaneous fully human monoclonal antibody directed against FXIIa, inhibiting its key proteolytic activity and the transformation of prekallikrein into kallikrein [53]. The first clinical trial for this drug was initiated in 2017 [54], and the phase 2 trial showed that treatment with this drug reduced breakthrough attacks by 98%-100% in comparison with the placebo and a favorable safety profile [55^▪▪^,^56^]. A phase 3 study (VANGUARD) confirmed the efficacy and safety of this drug at a loading dose of 400 mg and as a subcutaneous monthly injection of 200 mg, with the treatment group showing a lower monthly attack rate than the placebo group (0.27, 95% CI: 0.05–0.49 in the treatment arm vs. 2.01, 95% CI: 1.44–2.57 in the placebo arm) [55^▪▪^]. The proportion of patients who experienced no attacks during prophylaxis was 72% and 69% at 3 and 6 months, respectively. In addition, data from a phase 2, randomized, open-label extension study showed that garadacimab 200 mg/month for more than 2 years was well tolerated and consistently reduced the attack rate, improving QoL [57^▪▪^]. These findings provide evidence for the potential of this drug as a prophylactic treatment for HAE in adults and adolescents [55^▪▪^].

Donidalorsen is a subcutaneous antisense oligonucleotide drug designed to inhibit the production of plasma prekallikrein through ribonuclease H1-mediated degradation and inhibition of hepatic PK synthesis. A multicenter, randomized DBPC trial investigated the effectiveness and safety of monthly donidalorsen (80 mg) in patients with HAE. The phase 1 trial showed that the highest reduction in the plasma prekallikrein level was achieved at 80 mg, with no safety issues [58]. The percentage of patients assigned to the donidalorsen group who were attack-free was 92% during weeks 5–17 and 9–17, and 100% during weeks 13–17 of this study [58]. The phase 2 trial showed a 96% reduction in HAE attacks with no additional safety signals [59]. The phase 3 study OASIS-HAE assigned patients with HAE to receive donidalorsen (80 mg subcutaneously) or placebo once every 4 or 8 weeks to evaluate the reduction in attack rate from week 1 to week 25. In this study, the mean attack rate from week 1 to week 25 was 81% lower (95% CI, 65–89) in the 4-week group than in the placebo group (*P* < 0.001) and 55% lower (95% CI, 22–74) in the 8-week group than in the placebo group (*P* = 0.004). The median reduction in the attack rate from baseline was 90% in the 4-week group, 83% in the 8-week group, and 16% in the placebo group. The mean attack rate during weeks 5–25 was 87% lower (95% CI, 72–94) in the 4-week group than in the placebo group (*P* < 0.001), and 60% lower (95% CI, 25–79) in the 8-week group than in the placebo group. At week 25, donidalorsen administration every 4 weeks resulted in an improvement in the least-squares mean total score for the change in the Angioedema QoL Questionnaire (scores range from 0 to 100, with a score of 100 indicating the worst possible QoL) that was 18.6 points (95% CI, 9.5–27.7) better than that with placebo (*P* < 0.001) [60]. The OASIS-Plus open-label extension study evaluated the safety and efficacy of prolonged donidalorsen treatment following the phase 3 OASIS-HAE study. This ongoing study evaluated the safety and efficacy of long-term administration of donidalorsen every four weeks in patients previously treated with another LTP.

One of the most recently developed drugs for the treatment of HAE is the oral deucrictibant (PHA-022121). A recent phase 2 trial (CHAPTER-1) evaluated this drug as a potential agent in prophylactic therapy for HAE, and the findings showed that deucrictibant significantly reduced the rate of moderate and severe HAE attacks at doses of 20 and 40 mg/day. It was found to be well tolerated and reduced the occurrence of attacks treated with on-demand medications [61^▪^].

## CONCLUSION

For several years, the options for HAE treatment have been limited to drugs with substantial adverse effects, insufficient efficacy, and difficult administration routes. The implementation of newer therapies has facilitated the application of individualized action plans with the goal of minimizing the number of attacks and addressing the complex needs of patients. Moreover, switching from one treatment to another due to insufficient efficacy and/or adverse events has become easier. Over the next few years, disease control up to attack-free remission may become possible.

## Acknowledgements

*None.*

### Financial support and sponsorship

*None.*

### Conflicts of interest

*There are no conflicts of interest.*

## REFERENCES AND RECOMMENDED READING

Papers of particular interest, published within the annual period of review, have been highlighted as:

▪ of special interest
▪▪ of outstanding interest
